# Developing a Systems Biology Approach to Study Disease Progression Caused by *Heterodera glycines* in *Glycine max*

**Published:** 2007-06-05

**Authors:** Vincent P. Klink, Christopher C. Overall, Benjamin F. Matthews

**Affiliations:** 1 United States Department of Agriculture, Soybean Genomics and Improvement Laboratory, Bldg 006, Beltsville, MD 20705; 2 Department of Bioinformatics and Computational Biology, George Mason University, Manassas, VA 20110

**Keywords:** laser capture microdissection, soybean cyst nematode, microarray, RNA interference

## Introduction

The advent of molecular biology caused a reductionist “fever” to spread throughout the biological research community that continues to this day. The new molecular insights and techniques enabled researchers to probe the constituent parts of complex biological systems at unprecedented scale and detail. The reductionist approach naturally emerged: if we could now isolate and study the component parts of a system, we should be able to synthesize the information about the individual components into a unified understanding of the whole system. However, this “naive reductionism” (Bloom, 2001) needs to be balanced with a systems approach for a simple reason: the complex dynamics of a biological system often produce behaviors and properties that cannot be explained by the presence of a single component, but rather emerge from the interactions of the components of the system (so-called emergent properties).

Running counter to the molecular biology revolution, there were proponents of a systems-level approach to studying and understanding various types of complex systems, including those found in the social and biological sciences. The foundations of this approach can be found in the works of Bertalanffy (1976) and Wiener (1948). However, the lack of experimental and computational technology at the time hindered the adoption of a systems approach as a practical method of biological research and was thus overshadowed by the exciting and promising reductionist approach afforded by the new molecular biology techniques. After many years of exciting discoveries involving molecules in isolation, many in the research community realized that although this type of research is necessary to understand a biological system, it is not sufficient. Fortunately, the experimental and computational technology required for a more integrated approach to studying complex biological systems is finally catching up. Computers have become smaller, faster, and capable of storing vast amounts of data. Coupled with computer-assisted automation techniques, several high-throughput experimental technologies have emerged that enable a systems approach to biology, including DNA sequencing platforms (genome and EST), EST libraries, transcriptional profiling technologies (various microarray platforms), two-hybrid screening, protein microarrays, and tandem mass spectrometry, to name a few. These new techniques generate huge amounts of genomic, transcriptomic, proteomic, and metabolomic data that needs to be stored, accessed, and mined. Fortunately, computers can store these large amounts of data now and database technology has flourished and matured in recent years (Pandey and Mann, 2000; Lockhart and Winzeler, 2000; Ge et al. 2001; Grigoriev, 2001; Walhout and Vidal, 2001; Vidal, 2001; Jansen et al. 2002; Walhout et al. 2002; Gunsalus et al. 2004). Superseding the ability to *store* the data, the emergence of the World Wide Web (WWW) has made it possible to make biological data publicly available to the masses. The conjunction of database technologies and the WWW has given rise to publicly available nucleotide and protein sequence databases (i.e. GenBank, UniProt, ExPASy), biomolecule structure databases (PDB), metabolic network databases (i.e. KEGG), genetic databases (i.e. WormBase), annotation databases (i.e. gene ontology [GO]), and many others.

The first stage of a systems approach to studying an organism or biological phenomena is typically *discovery-based*, during which researchers characterize and catalog, in a high-throughput manner, the component biomolecules of the various levels of a system and their possible interactions. Functional and comparative genomics exist at the core of this research stage, whereby the genes, their encoded proteins, and gene regulatory interactions are identified for the biological system under study. Genome sequencing, creation of EST libraries, transcription profiling, and tandem mass spectrometry are typical activities during this stage and the wealth of knowledge obtained using reductionist approaches is invaluable for informing and fine-tuning the characterization of biomolecules. The second stage of the systems approach to studying an organism or biological phenomena is typically *hypothesis-based*, during which researchers determine functionally relevant biomolecules and interactions, develop models to explain and predict system behavior, and perform perturbation experiments to validate hypotheses. Although discovery-based research and hypothesis-based research are distinct and complementary research modalities in systems biology, it is important to note that the technologies and techniques often overlap. For example, microarrays are often used during discovery-based research to identify unknown gene functions (via clustering of expression profiles perhaps), but they are also used during hypothesis-based research to test hypotheses about system responses to various perturbations. This has been encountered in systems biology approaches to understand plant cell signaling (reviewed in Provart and McCourt, 2004) and the incorporation of protein data to study plant biology (reviewed in Glinski and Weckwerth, 2006). As our research into plant-pathogen interactions progressed using EST library analysis and microarrays, we found that we were implementing a systems biology approach and developing systems biology tools. A systems approach identifies genes important for biological processes (Janes et al. 2005; Janes and Yaffe, 2006; Albeck et al. 2006; Forst, 2006) and has been useful for our analysis of an important plant-pathogen interaction. It was then essential to develop databases that could manage those data.

## Significance of the problem

**Figure 1:**
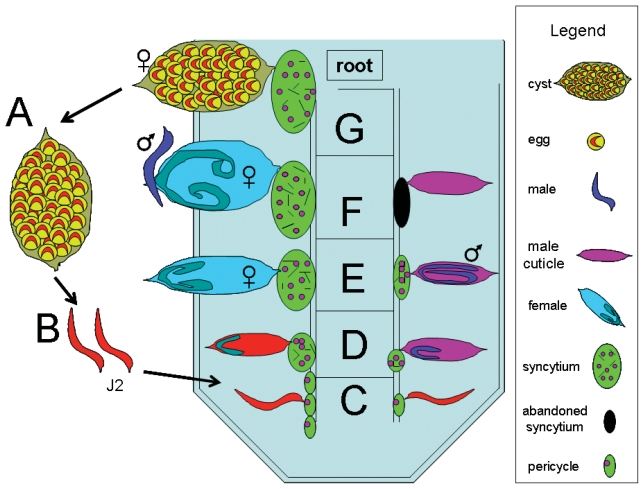
Life cycle of *H. glycines*. The life cycle of *H. glycines* is approximately 30 days and consists of three major stages, (1) egg, (2) mobile juvenile (J2) that undergoes successive molts (J3, J4) while within the root, and (3) adult that is either mobile (male) or sedentary (female). The egg, within cysts that can lie dormant in the soil for years, hatch as second stage juveniles (J2s) and migrate toward and subsequently penetrate a root. Once in the root, nematodes burrow to the root stele. A stylet emerges from the anterior end of the nematode and injects substances into a pericycle or neighboring root cell. The nematode, presumably, releases substances that then causes major changes in the physiology of the root cell. Those root cells then fuse with neighboring cells. This process transforms the fused root cells into a syncytium, a structure that ultimately contains ~200 merged root cells, and acts as the *H. glycines* feeding site for the balance of its lifecycle. During this time, the male nematodes feed for several days until the end of the J3 stage meanwhile becoming sedentary and exhibiting muscle degradation. Shortly afterwards, the males discontinue feeding, and molt into vermiform J4. They reactivate their muscles, emerge through the J2 and J3 cuticle and burrow toward the female to copulate. In contrast, the female becomes and remains sedentary once their feeding site is established. The female nematodes expand circumferentially and undergo J3 and J4 molts during feeding. They subsequently mature into feeding adult females. Ultimately, the female becomes the cyst that encases the eggs. Fig. 1A, cysts may lie dormant in the soil for years. Fig. 1B, second stage juveniles (red) hatch and migrate toward the root of *G. max*. Fig. 1C, J2 nematodes burrow into the root and migrate toward the root vasculature and select a cell for feeding site establishment. Fig 1D, J2 nematodes have molted into J3. Meanwhile, neighboring root cells have merged to form syncytia. Fig. 1E, Male and female J3 nematodes have molted into J4. Meanwhile, the female continues to grow circumferentially as it feeds. The male discontinues feeding at the end of its J3 stage. Fig. 1F, Male and female J4 nematodes become adults and the vermiform male migrates to the female to copulate. Fig. 1G, After ~30 days, the cyst is clearly visible, emerging from the root. Figure adapted from Jung and Wyss, (1999).

Parasitic plant nematodes cause billions of dollars in crop losses each year and cause problems universally in plants. The most economically devastating parasitic nematode is *Heterodera glycines* (soybean cyst nematode), a parasite that infects *Glycine max* (soybean). *H. glycines* is the major parasite of *G. max*. It causes approximately $460 to 818 million per year in losses in the U.S. (Wrather and Koenning, 2006). *H. glycines* invade roots, migrate to the stele and establish their feeding sites (Endo, 1965, 1991; Riggs et al. 1973; Kim et al. 1987) (Fig. 1). When susceptible (compatible reaction), roots fail to overcome infection and the nematode continues to feed and complete its lifecycle. During the resistance response (incompatible reaction), roots eventually overcome infection by localized changes in the syncytium and or the cells surrounding the syncytium (Endo, 1965, 1991; Riggs et al. 1973; Kim et al. 1987). Thus, it is apparent that specific events occur, making *G. max* hostile to *H. glycines* infection. There are numerous well-characterized *H. glycines* isolates that exhibit varying degrees of compatibility with *G. max* varieties (Riggs and Schmitt, 1991; Niblack et al. 2002). Resistance is limited to specific nematode races. Thus, resistance is not durable. These limitations necessitate ways to create *H. glycines* resistance in *G. max*.

Our lab studies the *G. max-H. glycines* plant-nematode system to understand how parasitic nematodes influence plant growth. The *G. max-H. glycines* system is used because resistant or susceptible reactions can be studied in the same *G. max* cultivar (i.e. Peking). The system is tractable for gene expression analyses (Cho et al. 2001; Zhang and Ghabrial, 2006). A new rapidly growing and dwarf *G. max* cultivar (MiniMax) has been generated, making a variety of genetic and molecular studies feasible (Matthews et al. 2007). The *G. max-H. glycines* system is also useful because information on genes identified in *G. max* can be translated directly to improve resistance. The development of several comparative databases (Alkharouf and Matthews, 2004; Alkharouf et al. 2004; Alkharouf et al. 2007) has allowed for a better understanding of emergent properties in both *G. max* and *H. glycines* occurring during infection.

## Historical techniques and research

### Histological analysis- historical perspective

**Figure 2:**
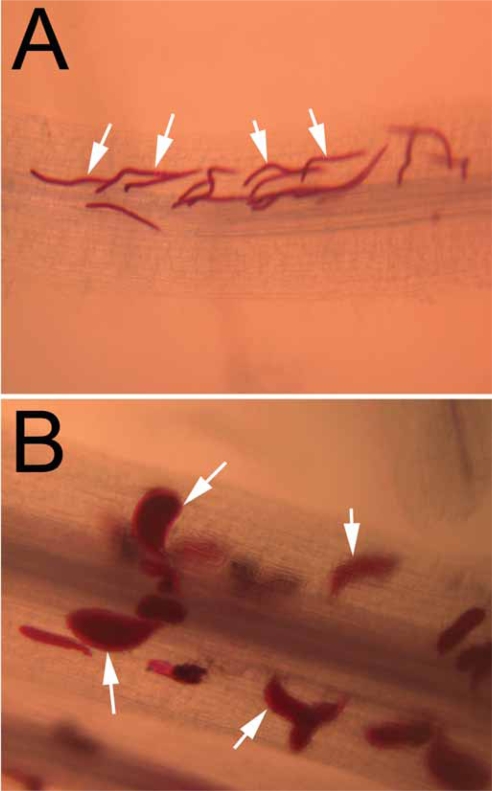
*G. max* cv. Peking seedlings were inoculated with *H. glycines* isolate TN8, resulting in a compatible reaction. Plants were harvested at three and eight dpi. Fig 2A, Peking infected with TN8 three dpi. Nematodes (white arrows) are stained red. Fig. 2B, Peking infected with TN8 eight dpi. Nematodes (white arrows) are stained red.

**Figure 3:**
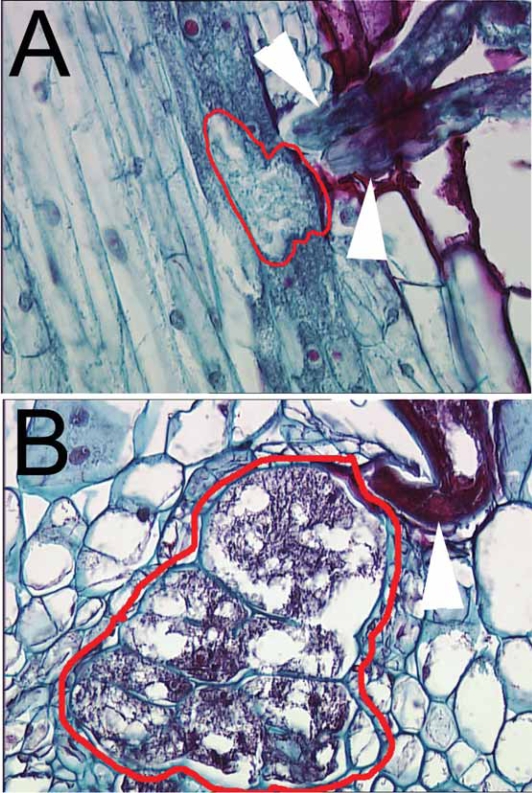
*G. max* cv. Peking seedlings were inoculated with *H. glycines* isolate TN8, resulting in a compatible reaction. Fig 3A, histological section of a root infected with TN8 (white arrowheads) at three dpi. The nematode feeding site (syncytium) has been encircled with a red line. Fig 3B, histological section of a root infected with TN8 (white arrowhead) at eight dpi. The nematode feeding site (syncytium) has been encircled with a red line.

There are numerous histological studies of the interactions between *G. max* roots and *H. glycines* (Endo, 1964, 1965, 1971, 1991; Endo and Veech, 1970; Gipson et al. 1971; Jones and Northcote, 1972; Riggs et al. 1973). These studies demonstrated the anatomical changes that occur in *G. max* roots during *H. glycines* invasion (Figs. 2A, B). During infection, *H. glycines* penetrate the root and migrate toward the vascular tissue. At approximately two days post-inoculation (dpi), the nematode selects a cell adjacent to the vascular tissue and pierces it with its stylet to initiate a feeding site or syncytium. The nematode presumably releases substances it synthesized in gland cells into the plant cell to initiate syncytium formation. Thus, the development of the syncytium is an emergent property of the infection process. By three days, the plant cell further enlarges (Fig. 3A). The cells adjacent to the feeding site become metabolically hyperactive (Endo and Veech, 1970; Endo, 1971). After a cell is selected and pierced by the nematode, the walls of the plant cells adjacent to the selected cell begin to dissolve. Eventually these recruited cells merge to form a syncytium or larger feeding cell (Fig. 3B). In susceptible roots, a number of changes occur to the cells at the nematode feeding site. The nuclei and nucleoli hypertrophy, cytoplasmic organelles proliferate, the central cell vacuole is reduced or lost, the cell expands, and the cell wall becomes perforated (Jones and Dropkin, 1975; Endo and Veech, 1970; Gipson et al. 1971; Riggs et al. 1973). The perforations enlarge as the cell expands and surrounding cells merge at the perforations to enlarge the feeding site. The girth of the feeding nematode becomes noticeably larger at four and six dpi and is especially noticeable at eight dpi.

In contrast, in the resistance response of *G. max* cultivar ‘Peking’ to *H. glycines* NL1-RHp, the syncytium collapses and becomes necrotic. Riggs et al. (1973) noted that cell wall depositions form and there is an increase in lipid globules before necrosis in Peking four dpi. There is also a concomitant degeneration of syncytia. Thus, a major mechanism of resistance may be to wall off the area of nematode invasion with a secondary wall to either starve the nematode or to allow toxic by-products to build up in the area. The resistant cultivar ‘Bedford’ has a somewhat different response, in that the nuclei degrade, then the cytoplasm degrades (Kim et al. 1987). Interestingly, syncytia form in both susceptible and resistant *G. max* genotypes, including PI 437654, which possesses broad resistance against *H. glycines* (Mahalingam and Skorupska, 1996). Certainly, development and maintenance of the syncytium is crucial to the survival of *H. glycines*. It is unclear if resistance is due to localized events or if these events occur throughout the root. Many pathways, genes and proteins are likely to be involved during the infection process.

### Gene expression-historical perspective

There are a few reports concerning the molecular aspects of susceptibility and resistance of plants to nematodes (Gurr et al. 1991; Cai et al. 1997; Grundler et al. 1997; Hermsmeier et al. 1998; reviewed in Williamson and Kumar, 2006). However, most discuss the expression of only one or a few genes (reviewed in Gheysen and Fenoll, 2002; Williamson and Kumar, 2006). Many labs have been using a variety of methods to better understand the problem of susceptibility. As will be mentioned below, these labs have focused on the identification of resistance (R) genes, candidate gene approaches, single gene analyses, and microarray analyses.

The cloning of R genes is important because of the potential to use those genes to engineer resistance. R genes (Jones et al. 1994; reviewed in Glazebrook, 2001, 2005) are plant genes that encode determinants for the activation of a plant defense response to pathogens. They have been shown to confer defense against bacteria and other pathogens such as the insect *Macrosiphum euphorbiae* (Rossi et al. 1998). Genes that actually confer resistance to nematodes are scarce (Williamson and Kumar, 2006). These R genes, identified by map-based cloning, include ones that defend against *Heterodera schachtii* (Cai et al. 1997) and *Meloidogyne javanica* and *M. incognita* (Milligan et al. 1998). It is not clear whether functional homologs exist in all agricultural plants. Resistance loci to *H. glycines* are present in the germplasm of *G. max*. These loci have been physically mapped (Cregan et al. 1999; Matthews et al. 2001). However, resistance is restricted to varieties that are the poorest with respect to production yield. In addition, resistance is limited to specific nematode populations within those varieties exhibiting resistance (Riggs and Schmitt, 1991; Niblack et al. 2002). Those R genes have not yet been identified.

Candidate resistance gene approaches have been performed (van der Vossen et al. 2000; Ernst et al. 2002; Paal et al. 2004). During this process, resistance genes from one plant are genetically engineered into another plant. These genes are then evaluated for their ability to confer resistance in the species it is introduced.

Single gene expression analyses have been performed. Numerous nematode-induced transcripts from tomato have been sequenced and identified (Bird and Wilson, 1994; Wilson et al. 1994). An understanding of the molecular response of *G. max* to *H. glycines* has been gained using differential display and northern blots. In these experiments, Hermsmeier et al. (1998) noted major changes in *G. max* gene expression in the susceptible interaction of *G. max* with *H. glycines*. Transcripts for five genes increased upon *H. glycines* infection, while transcripts of ten others decreased. Until recently, little was known about the molecular response of resistant and susceptible cultivars of *G. max* to *H. glycines*. Edens et al. (1995) examined the expression of some of the genes involved in phenylpropanoid synthesis. This is an important plant defense pathway. Transcription of the genes encoding phenylalanine ammonia lyase and 4-coumaryl CoA- ligase increased, as did these enzyme activities, in resistant, but not in susceptible cultivars of *G. max* after infection by *H. glycines*. However, transcription of genes encoding enzymes found later in the pathway was enhanced in both resistant and susceptible cultivars after nematode invasion.

Microarrays were originally developed for the study of plant gene expression (Schena et al. 1995) and used subsequently to study infection of plants by a pathogen (Schenk et al. 2000). Microarray analyses have been used to study nematode infection in the model plant *Arabidopsis thaliana* (Puthoff et al. 2003; Jammes et al. 2005). Puthoff et al. (2003) examined sugarbeet cyst nematode (*Heterodera schachtii*) and *H. glycines* interactions with *A. thaliana* using microarrays to measure gene transcript levels. There were 128 genes identified with altered gene transcript levels in *A. thaliana* roots after infection by the sugarbeet cyst nematode, while only 12 genes were identified with altered transcript levels following *H. glycines* infection. Jammes et al. (2005) identified thousands of genes exhibiting various profiles of expression during a time-course of *M. incognita* infection in *A. thaliana*. Microarray analyses focusing on the susceptible response have been performed in *G. max* (Khan et al. 2004; Alkharouf and Matthews, 2004; Alkharouf et al. 2004, 2005, 2006). For an in-depth review of nematode infection, see Gheysen and Fenoll (2002).

Studies investigating nematode gene expression have also been performed. These analysis methods include the isolation and identification of putative parasitism genes (Gao et al. 2001; Wang et al. 2001; Bekal et al. 2003; Lambert et al. 2005). A cDNA query database (i.e. http://www.nematode.net; Washington University, St. Louis) has also been developed. Databases that can be used to perform comparative analyses between different nematode species have also been generated (Alkharouf et al. 2007) that also demonstrated RNA in *H. glycines*.

The aforementioned approaches are the initial steps to understanding the complexity of the interaction between the plant and infectious nematodes. Methods that can integrate these data will help to better understand their emergent properties during infection. The following sections will expand and examine how both discovery-based techniques and research and hypothesis-based techniques and research are being merged to study *G. max* infection by *H. glycines*.

## Discovery-based techniques and research

### Database management tools

It is clear from the historical observations that differential gene expression in *G. max* and *H. glycines* occurs during resistance and susceptibility. To understand this problem better and to develop new solutions through biotechnology, a systems approach is required because in the plant, tissue and cell morphology is grossly altered, the levels of many different gene transcripts are altered, proteins are altered in abundance and activity, and metabolite concentrations are changed. The goal, therefore, is to find out what is changing and why it is changing during the course of infection.

One discovery method already mentioned is the use of microarrays. Microarray analysis of gene expression in *G. max* roots infected by incompatible or compatible isolates of *H. glycines* would likely identify genes preferentially expressed during resistance. An obstacle to analyzing the vast amounts of gene expression data is its organization, management and maintenance. New technologies have been developed that are now available to begin identifying and better understanding genes in both *G. max* and *H. glycines*. Once a large amount of *G. max* and *H. glycines* genes were available in GenBank, they could be annotated to better understand their role(s) in the biology of those organisms. This is one of the most basic organizational levels of identifying gene function. A gene ontology (GO) analysis (Ashburner et al. 2000; Gene Ontology Consortium, 2001) would permit a better understanding of both *G. max* and *H. glycines* (Alkharouf et al. 2007) genes. Once these analyses were made, those data could be centralized in a database and made available to researchers (Alkharouf and Matthews, 2004; Alkharouf et al. 2007).

### Soybean Genomics Microarray Database (SGMD)

The centralization of expression data in our lab has begun with the Soybean Genomics Microarray Database (SGMD). The Soybean Genomics Microarray Database (SGMD) is a resource to mine the gene expression data of thousands of genes identified by microarray analysis (Alkharouf and Matthews, 2004). Housed in the SGMD are the results of several microarray analyses. These analyses include a time-course microarray analysis of 6,500 genes using seven time-points during the course of a susceptible interaction (Alkharouf et al. 2006). This database has since been supplemented with several other microarray experiments and genomic data. The online analytical processing (OLAP) capabilities of the SGMD database are another resource that can be used to query those data (Alkharouf et al. 2005). This allows scientists, who have little or no knowledge of bioinformatics, to mine the microarray database without downloading the data and using third-party analysis software.

### CeHg database-gene identification in *H. glycines*

Data on genes often reside in non-connected databases and thus impede contextual analyses. The isolation of cDNAs important to *H. glycines* parasitism (Gao et al. 2001; Wang et al. 2001) was essential for an understanding of the earliest stages of parasitism (Bakhetia et al. 2007). This is because it provided a set of genes that encode proteins that are presumably injected by the nematode into plant cells during infection. Those *H. glycines* gene sequences are available at both GenBank and http://www.nematode.net. The availability of those sequences then allowed for a comparative analysis. However, those data exist in unlinked databases, making comparative analyses seemingly intractable. The *Caenorhabditis elegans*-*Heterodera glycines* (CeHg) database (Alkharouf et al. 2007) is a comparative analysis of *H. glycines* with *C. elegans*, a nematode with a fully-sequenced and well-characterized genome. The linking of those databases was essential to organize, manage and maintain the data. It was also essential so that interfaces with other databases (i.e. Wormbase.org) could be created. In that analysis, over 300,000 gene sequences from *C. elegans* were compared with the EST sequences of *H. glycines* (Alkharouf et al. 2007). Those analyses identified approximately 8,000 gene sequences that were conserved between *H. glycines* and *C. elegans*. The *C. elegans* database (Wormbase.org) also provides a wealth of information concerning genetic mapping data and phenotypes identified by mutational analysis by a large community of researchers. Another resource available in Wormbase.org is RNA interference (RNAi) data for thousands of genes. RNAi (Fire et al. 1998) was originally identified as a means to eliminate the endogenous pool of cognate RNA for a particular gene. RNAi is a process by which double stranded RNA is introduced into a cell (or organism) and, by a well-defined biochemical process, the cognate RNA normally produced in the cell is degraded while other RNAs are not affected (Fire et al. 1998). Because the RNAi outcome typically mimics the mutant phenotype of that gene, the result is called a phenocopy. The analysis of *H. glycines* gene sequences was then expanded by putting an additional constraint on the analysis. All of the conserved genes that occur between *H. glycines* and *C. elegans* (from the current gene sequence data available in GenBank) were identified that also had RNAi experimental data (in Wormbase.org) that demonstrated that the *C. elegans* phenocopy was lethal. It was believed that the RNAi constraint would maximize the probability of identifying *H. glycines* genes whose RNAi phenocopies were lethal. That analysis was followed up by RNAi studies that demonstrated RNAi for a putative lethal candidate gene resulted in lethality for *H. glycines*, thus demonstrating the efficacy of the approach (Alkharouf et al. 2007). Other analyses comparing *H. glycines* with *C. elegans* will undoubtedly aid in the understanding of its pathogenicity and will allow for the development of biocontrol measures. Those comparisons will be aided by identifying active metabolic pathways whose constituent genes (and thus their corresponding proteins) are induced or suppressed on microarrays during the course of a biological process. These comparisons will be aided by applications such as ResNet and PathwayStudio (Ariadne Genomics) and Kyoto Encyclopedia of Genes and Genomes (KEGG), (Kanehisa Laboratories, Kyoto University and the Human Genome Center, University of Tokyo). These interfaces allow for the functional categorization of genes presently managed in disparate databases.

### Whole root genomic analysis of *G. max*

Alkharouf et al. (2004) examined the development of an infection process by studying the gene transcript levels of ~6,500 genes in *G. max* roots susceptible to *H. glycines* two dpi., Because syncytia are formed by *H. glycines* at approximately 1.5 to two dpi, RNA was isolated from *G. max* roots two dpi. This allowed the examination of transcript levels during the susceptible response at this critical time point. That analysis identified a number of genes having elevated and suppressed transcript levels. Approximately 8% of the genes represented on the microarray were induced. Noteworthy genes included those encoding the repetitive proline-rich glycoprotein, SAM22, peroxidase and those involved in plant defense and signaling. Interestingly, the function was unknown for over 50% of the induced genes.

Alkharouf et al. (2006) expanded on that study by including time-points at six, 12 and 24 hours post-inoculation (hpi), two, four, six and eight dpi. Therefore the *development* of a susceptible infection was analyzed. It takes at least 18 to 24 hours before most of the nematodes reach cells near the vascular tissue to initiate a feeding site. Prior to these times, the nematodes are burrowing into the root, but have not started to feed yet. Thus, a significant part of that experiment was the inclusion of time-points occurring before the nematode establishes its feeding site. Those time-points allowed for the determination that the plant responds to the nematode as soon as the nematode is burrowing through the root. Numerous genes were induced at the early time points of six and 12 hpi during *G. max* infection by *H. glycines*. Some of those genes were well-known genes involved in the defense response. Those genes included Kunitz trypsin inhibitor SAM22, phospholipase D and 12-oxyphytodienoate reductase. Those results suggested that there are important changes in gene expression occurring during the susceptible response while the nematode is burrowing through the root and before they begin feeding. RT-PCR was used to confirm the expression of some of the genes, including calmodulin, trehalose phosphate synthase, lipoxygenase, phospholipase C, chalcone reductase and WRKY6 (Alkharouf et al. 2006).

The identification of the induction of WRKY gene expression during the susceptible reaction was particularly intriguing. This is because WRKY transcription factors are involved in the defense response (Shen et al. 2007). Recent microarray analyses have demonstrated WRKY gene expression during infection of *A. thaliana* by the bacterium *Pseudomonas syringae*. Those analyses demonstrated that the activation and downstream targets of the WRKY gene family is complex (Wang et al. 2006). Those microarray analyses have aided their understanding of the activation of gene networks during infection (Wang et al. 2006). WRKY genes have been shown to be rapidly induced during the resistance response in pepper (*Capsicum annum*) (Oh et al. 2006). *A. thaliana* mutants of WRKY33 become susceptible to the fungal pathogens *Botrytis cinerea* and *Alternaria brassicicola* (Zheng et al. 2006). However, over-expression of WRKY33 was shown to enhance susceptibility to *P. syringae* and is coincident with reduced PR1 gene expression (Zheng et al. 2006). Ryu et al. (2006) explored the expression of 45 WRKY genes in *Oryza sativa*. They demonstrated that 15 WRKY genes were significantly induced during the resistance response after infection with the fungus *Magnaportha grisaei* (Ryu et al. 2006). Twelve of those WRKY genes were also induced during infection with the bacterial pathogen *Xanthomonas oryzae* pv. oryzae (Xoo) (Ryu et al. 2006). Xu et al. (2006) demonstrated that, in *A. thaliana*, WRKY18, WRKY40 and WRKY60 form a multimeric complex. The influence of WRKY gene expression during resistance to *P. syringae* and *B. cinerea* was tested by mutational analysis. Single mutants of wrky18, wrky40 or wrky60 had little effect (Xu et al. 2006). However, wrky18-wrky40 and wrky18-wrky60 double mutants exhibited resistance to *P. syringae*, but were susceptible to *B. cinerea* (Xu et al. 2006). The wrky18-wrky40-wrky60 triple mutant was also resistant to *P. syringae* and susceptible to *B. cinerea* (Xu et al. 2006). Enhanced disease resistance was also observed in *A. thaliana* wrky11 and wrky17 single mutants and enhanced resistance was observed in wrky11-wrky17 double mutants infected with *P. syringae* (Journot-Catalino et al. 2006). The WRKY gene family is also large in *G. max*. It is likely that complex gene expression patterns will emerge as we better understand *G. max* infection by *H. glycines*.

Later time-points during the infection of *G. max* by *H. glycines* were also studied. At the later time-points of six and eight dpi, *H. glycines* has found a potential feeding site and punctured a cell with its stylet. The emergent reaction is the formation of the syncytium. At these later times the transcripts were elevated for numerous genes involved in transcription and protein synthesis, carbon metabolism and transport, including sucrose synthase and glyceraldehydes 3-phosphate dehydrogenase. The generation of these data during the discovery phase of research has provided a useful resource that can now be further analyzed so that the expression data can be put into a functional context using applications such as Metacyc that link microarray expression data to metabolic pathways (Caspi et al. 2006).

### Analysis of specific cell types of *G. max* and *H. glycines*

Important molecular interactions leading to resistance have been shown to occur in other plants within the specific cell types experiencing those interactions. For example, Shen et al. (2007) demonstrated how the interaction between a fungus (*Blumeria graminis*) avirulence effector (*AVR_A10_*), the barley *mildew A* (*MLA*) *R locus* (MLA10) and WRKY transcription factors were important to resistance in the specific plant barley cells that contain them (Shen et al. 2007; reviewed in Sheen and He, 2007). This may relate to *G. max* infection by *H. glycines* infection because R genes have been shown to confer resistance in plants to other nematode species (Cai et al. 1997; Milligan et al. 1998; van der Vossen et al. 2000; Ernst et al. 2002; Paal et al. 2004). The involvement of R genes and WRKY genes during *G. max* infection by *H. glycines* may work in some manner analogous to those observed in barley (Shen et al. 2007). Clearly, there are important interactions occurring between *G. max* and *H. glycines* within the syncytium. Thus, isolating syncytia from the surrounding tissue may provide clues as to how the cell emerges and develops. It may also help us understand how the syncytium is sustained by the plant (or coerced by the nematode) throughout the course of nematode development.

**Figure 4:**
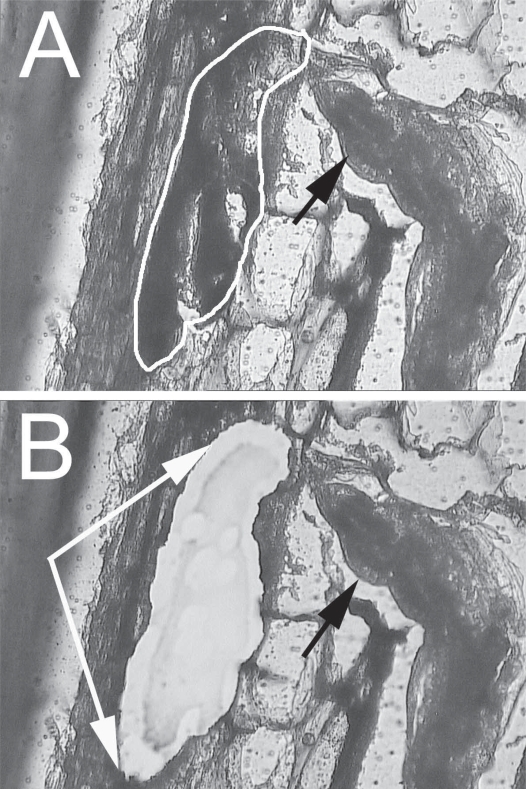
Syncytia from *G. max* were microdissected for gene expression experiments. Fig. 4A, an eight dpi time-point syncytium (arrow) prior to microdissection was identified by their proximity to the anterior end of *H. glycines* (black arrow). Fig. 4B, the same syncytium section from Fig. 4A after microdissection (area between arrows). The black arrow points toward the nematode.

A method to isolate cells from complex tissues is Laser Capture Microdissection (LCM). LCM was first developed and used to study animal cells (Meier-Ruge et al. 1976; Isenberg et al. 1976). LCM, thus, allowed them to overcome problems with hand-microdissection of tissues. LCM was further developed to accommodate paraffin-embedded samples on microscope slides (Emmert-Buck et al. 1996) and was first used by Asano et al. (2002) in plants. LCM can be used to isolate syncytial cells formed by the feeding nematode (Fig. 4) (Klink et al. 2005). RNA can be extracted from these isolated feeding cells and used to construct a cDNA library for EST analysis (Klink et al. 2005). Sequence analysis of a cDNA library can provide information on gene identity and provide a representation of the transcript abundance of genes expressed in the syncytium. The analysis of sequenced ESTs from the feeding site cDNA library identified numerous genes, including genes encoding tubulin, several ribosomal proteins, DNA-binding proteins and transcription factors, a protein kinase, two peroxidases, a protease, aquaporin, carbonic anhydrase, and other proteins. The extracted RNA also can serve as a template to perform gene expression experiments (Nakazono et al. 2003). Transcript levels of aquaporin (GmPIP2,2), α-tubulin, β-tubulin and genes of unknown function were elevated in syncytial tissue (Klink et al. 2005). Full-length clones of GmPIP2,2 and α-tubulin were identified and sequenced (Klink et al. 2005). Transcripts of GmPIP2,2 were localized by *in situ* hybridization to cells immediately adjacent to the syncytium, while immuno-histocytochemistry was used to demonstrate α-tubulin localization to the syncytium (Klink et al. 2005).

The coercion of *G. max* by the nematode probably occurs via substances that are injected into the plant. The dorsal esophageal gland cell and the subventral esophageal gland cells are the sites of synthesis of proteins that presumably are injected through a piercing stylet mouthpiece of the nematode into plant cells. Methods have been employed to isolate those cell specific cDNAs in *H. glycines* (Gao et al. 2001; Wang et al. 2001). Consistent with this was the isolation and characterization of one of these nematode genes having homology to an important plant protein involved in various aspects of growth (Clark et al. 1996; Wang et al. 2005). The isolation of cDNAs from the syncytium (Klink et al. 2005) along with the isolation of cDNAs from glands important for *H. glycines* pathogenicity (Gao et al. 2001; Wang et al. 2001; Bakheita et al. 2007) are allowing for the isolation and subsequent analysis of cDNAs expressed in the actual cell types important to infection.

## Hypothesis-based techniques and research

It is essential to find G. *max* and *H. glycines* genes that may be relevant to the infection process. This occurs during the discovery phase of the research. The hypothesis-based phase of research required the development and utilization of certain technologies and methodologies that allowed for the perturbation of the normal function of these genes. Putative candidate lethal *H. glycines* genes have been identified by comparative analyses (Alkharouf et al. 2007). This will allow for the identification of their actual role(s) during infection. The following sections describe the technologies and methodologies developed to study those genes.

### Database management as a means to drive hypothesis-based techniques and research

The sequencing of the *C. elegans* genome (The *C. elegans* Sequencing Consortium, 1998) and the *A. thaliana* genome (Arabidopsis Genome Initiative, 2000) paved the way for a systems approach to understand their development. Genomic RNAi screens (Sonnichsen et al. 2005) housed in the updated Wormbase (Bieri et al. 2007) provide a large amount of data to manage. A limitation encountered during the study of *C. elegans* was the development of ways to manage those large databases. In addition, it became important to have ways to link data obtained in these *C. elegans* RNAi studies (phenome mapping) to data generated from microarrays (transcriptome) and protein-protein interaction (interactome) (Walhout et al. 2002). Some of these problems were solved by the development of RNAiDB and PhenoBlast tools (http://www.rnai.org) that permits the user access to time-lapse movies and a variety of other data pertaining to RNAi experiments (Gunsalus et al. 2004). Many of these and other limitations had been solved originally in *Saccharomyces cerevisiae* (Pandey and Mann, 2000; Lockhart and Winzeler, 2000; Ge et al. 2001; Walhout and Vidal, 2001; Vidal, 2001).

### RNAi studies- *H. glycines*

One way to study infection is to study genes essential for the pathogen to function. A method showing much promise is the production of transgenic plants that produce RNAi constructs for genes essential to pathogen viability. The plant will not be affected by RNAi constructs engineered for pathogen genes. This is due to the gene specific nature of RNAi. The premise is that the pathogen will somehow imbibe the dsRNA that is produced within the host (Winston et al. 2002; Feinberg and Hunter, 2003). Once the dsRNA is within the cells of the pathogen the RNAi pathway will be engaged. If the gene is essential, the pathogen will lose viability over time as the cognate RNA pool is depleted and its protein content degrades. Early efforts using this strategy were successful. For example, RNAi has been used to study genes essential for infection of *A. thaliana* and *Lycopersicon esculentum* by *Agrobacterium tumefaciens* (Escobar et al. 2001). Infection of plants by *A. tumefaciens* is a two-step process. Firstly, the bacterium performs a horizontal gene transfer and integration into the plant genome (transformation). Secondly, plant cell division is engaged, resulting in the formation of a crown gall (tumorigenesis). This occurs as a consequence of gene activation on the horizontally transferred DNA sequence (Escobar et al. 2001). Escobar et al. (2001) generated plants transgenic for *A. tumefaciens iaa*M and *ipt* genes. The *iaa*M gene encodes tryptophan monooxygenase (Klee et al. 1984) while *ipt* encodes a gene whose product catalyzes the condensation of AMP and isopentyl pyrophosphate to produce zeatin, a cytokinin (Lichtenstein et al. 1984). These two genes reside on the tumor-inducing (Ti) plasmid of *A. tumifaciens* and are essential for wild-type tumor formation (Ooms et al. 1981). Escobar et al. (2001) demonstrated that plants transgenic for an RNAi construct that generated dsRNA for both *iaa*M and *ipt* inhibited crown gall formation. This provided a model for the use of RNAi in the study and control of other plant pathogens.

RNAi has been used in candidate approach investigations to study *H. glycines* genes (Urwin et al. 2002; Alkharouf et al. 2007; Bakheita et al. 2007). Urwin et al. (2002) employed a candidate gene approach investigating cysteine proteinases, C-type lectins and major sperm protein. Cysteine proteinase RNAi experiments reduced the male/female ratio while RNAi for C-type lectins decreased the number of nematodes. RNAi for the major sperm protein had no effect (Urwin et al. 2002). Bakheita et al. (2007) focused primarily on genes whose products are secreted substances that subvert the growth of the plant. Other ways to study the process of infection is to study the role of genes that are essential to viability or the development of *H. glycines* as the J2s develop into mature adults or the development of embryos within the gravid females. One limitation would be in identifying large numbers of essential genes by candidate gene approaches. After the discovery of RNAi in *C. elegans* (Fire et al. 1998), researchers realized the power that large-scale genomic screens could have in identifying the function of a large number of genes important for viability and development (Piano et al. 2000; Sonnichsen et al. 2005). In addition, RNAi would be of particular use to study genes that supply maternal pools of RNA that are essential for embryogenesis (Piano et al. 2000). These studies demonstrated the efficacy of the approach to applying large-scale screens to identify genes essential for specific biological functions.

The identification of putative lethal genes in *H. glycines* became simplified by the various available *C. elegans* RNA databases. However, the identification of genes was still confounded by the data residing in disparate databases. The CeHg database has been developed and used to identify ~1500 genes that could potentially yield lethal phenocopies in *H. glycines* (Alkharouf et al. 2007). Those analyses have identified numerous genes that perform roles in development, metabolism, cell structure, protein synthesis, transport and DNA synthesis (Alkharouf et al. 2007). Those predictions have been tested by performing RNAi experiments of *H. glycines* genes (Alkharouf et al. 2007). In one of those tests, a lethal phenocopy of a gene shown to be essential for viability was obtained (Alkharouf et al. 2007). Other uses of the CeHg database have been the identification of *H. glycines* homologs of genes involved in muscle composition and maintenance in *C. elegans* (Klink et al. 2007). These studies have demonstrated a decrease in transcript abundance of several genes that perform important roles in muscle biology, presumably as a consequence of the sedentary phase of their lifecycle (Klink et al. 2007). Those hypothesis-based analyses of *H. glycines* gene function demonstrate that large numbers of genes can be rapidly screened as a means to better understand important aspects of *H. glycines* development. These analyses have also identified numerous genes of unknown function. Those genes could be the focus of RNAi screens to identify their function during parasitism. Some of these unknown genes were originally isolated from gland cells known to be important for parasitism (Gao et al. 2001; Wang et al. 2001). Some of these putative parasitism genes have subsequently been tested through knock down RNAi analyses in *H. glycines* (Bakheita et al. 2007). Experiments have shown in other systems that expressing nematode genes as RNAi constructs in plants can be used as a biocontrol for parasitic nematodes (Huang et al. 2006). Using such strategies should prove promising in better understanding genes essential for parasitism (Bakheita et al. 2007) and those important for various aspects of development of *H. glycines* (Alkharouf et al. 2007).

### MiniMax as a model for soybean research

A major limitation to test the emergent properties of genes in *G. max* research is the time it takes to grow and engineer genes into them (Bailey et al. 1993; Santarem and Finer, 1999; Ko et al. 2004; Schmidt et al. 2005). Researchers have used dwarf varieties of agricultural plants as an avenue that can be taken to avoid the problem of plant size and generation time (Scott and Harbaugh, 1989; Meissner et al. 1997). Recently, a dwarf variety of *G. max* that has the additional benefit of growing rapidly has been developed as an alternative variety for genetic and molecular research (Matthews et al. 2007). This variety, called MiniMax, has several features (i.e. the ability to be transformed, rapid generation time, compact size) that make it especially useful for laboratory research. MiniMax can be used to test the roles of genes attributed to emergent properties because of its ability to be genetically transformed.

### Mutagenic analyses

Chemically induced mutagenesis can and has been used to generate mutations useful for determining gene function in plants and animals (reviewed in Schimenti and Bucan, 1998). The feasibility of using ethyl methanesulfonate (EMS) to generate a range of mutations using MiniMax has been tested. The combination of small size and rapid generation time makes MiniMax an ideal cultivar for hypothesis-based systems-level analysis, enabling thousands of parallel experiments (mutation analysis) to be performed in a shorter period of time while studying *G. max* responses to *H. glycines* infection. Mutations in height, structure and fruit set have been identified, suggesting that useful mutations could be found using chemical mutagenesis of MiniMax.

An application of mutagenesis screening is targeting induced local lesions in genomes (TILLING). TILLING is a high-throughput technique that allows the correlation of gene mutation with phenotype. McCallum et al. (2000a, b) used EMS to generate a population of mutagenized *A. thaliana* plants for TILLING. In addition to *A. thaliana* Benaben et al. (1995) developed EMS mutants of *Medicago truncatula*, Perry et al. (2003) developed a collection of EMS mutants of *Lotus japonicus,* and Till et al. (2004) established a collection of point mutations in maize which he screened using TILLING.

As mentioned earlier, R genes have been identified in other plants to confer resistance to parasitic plant nematodes (Cai et al. 1997; Milligan et al. 1998; Hwang et al. 2000; van der Vossen et al. 2000; Ernst et al. 2002; Hwang and Williamson 2003; Paal et al. 2004). Because many R genes have been identified in *G. max* by a variety of screening methods (Graham et al. 2002 Graham et al. 2003; Peneuela et al. 2002; He et al. 2002; Ashfield et al. 2003), mutated forms of those genes could be produced and identified via TILLING. Their emergent properties in nematode resistance could then be tested with simple visual screens.

### Plant transformation

Plant transformation can be used to study the emergent properties occurring during the *G. max* interaction with *H. glycines*. Using plant transformation, certain genes are over-expressed or silenced to better understand their function. Over-expressing and/or silencing R genes in *G. max* could be another way to identify the potential roles of those genes in *G. max*. Transformed *G. max* root cultures can be rapidly produced by using *Agrobacterium rhizogenes* mediated hairy roots (Savka et al. 1990; Cho et al. 2001). Thus, they can be used for large-scale genetic screens. Alternatively, viral expression systems can be used to overexpress genes or be used for virus induced gene silencing (VIGS) to silence genes in *G. max* (Zhang and Ghabrial, 2006). The application of MiniMax is an especially promising development because it will allow rapid testing of the predicted functionality of individual genes as well as emergent functionality resulting from the combined effects of multiple genes of the host and its pathogen. The dwarf nature of MiniMax permits the study of large numbers of these compact plants in a small space.

## Future directions

Over the course of the past few years, a systems-level approach has overcome several limitations inherent in the traditional *G. max-H. glycines* research paradigm. The manufacture and use of *G. max* cDNA microarrays has allowed the study of the expression patterns of thousands of *G. max* genes simultaneously at various times during the *H. glycines* infection process. Those analyses led to the identification of several genes involved in defense pathways in other organisms. The isolation of homogeneous cell populations using LCM has allowed for profiling the transcriptome of the feeding site of the nematode. The development of MiniMax, a dwarf, transformable and rapidly growing *G. max* variety should allow researchers to overcome space and time limitations of standard *G. max* varieties. The compilation of these data, using a variety of interconnected databases, allows for the identification of active sub-networks from gene expression data. The implementation of new database search techniques will allow for the correlation of gene expression data with the available data on gene pathways (Metacyc [Caspi et al. 2006] and other resources). This will allow for the dissection of how members of genes and gene families function during infection (Wang et al. 2006). Such analyses may aid in the identification of their function during resistance of *G. max* to *H. glycines.*
